# Supplementary material for: Cervical length varies considering different populations and gestational outcomes: Results from a systematic review and meta-analysis
Source: PLoS One. 2021 Feb 16;16(2):e0245746. doi: 10.1371/journal.pone.0245746 (PMC7886126; doi:10.1371/journal.pone.0245746)
Supplement: S2 Appendix — (DOCX) [file pone.0245746.s002.docx]

**S2 Appendix - Database research: search syntaxes**

Medline:

((((((((((((((((((((Ultrasonography, Prenatal[MeSH Terms]) OR Prenatal Ultrasonic Diagnosis[MeSH Terms]) OR Prenatal Ultrasonography[MeSH Terms]) OR Diagnosis, Ultrasonic Prenatal[MeSH Terms]) OR Diagnoses, Ultrasonic Prenatal[MeSH Terms]) OR Prenatal Diagnoses, Ultrasonic[MeSH Terms]) OR Ultrasonic Prenatal Diagnoses[MeSH Terms]) OR Ultrasonic Prenatal Diagnosis[MeSH Terms]) OR Diagnosis, Prenatal Ultrasonic[MeSH Terms]) OR Diagnoses, Prenatal Ultrasonic[MeSH Terms]) OR Prenatal Ultrasonic Diagnoses[MeSH Terms]) OR Ultrasonic Diagnoses, Prenatal[MeSH Terms]) OR Prenatal Diagnosis, Ultrasonic[MeSH Terms]) OR Ultrasonic Diagnosis, Prenatal[MeSH Terms]) OR Fetal Ultrasonography[MeSH Terms]) OR Ultrasonography, Fetal[MeSH Terms])) OR (((((((“screening”) OR “transvaginal ultrasound”) OR “ultrasound”) OR “ultrasound screening”) OR “second trimester ultrasound”) OR “vaginal sonography”) OR “transvaginal ultrasound”))) AND ((((((((“preterm parturition”) OR “prematurity”) OR “preterm labor”) OR “preterm delivery”) OR “preterm birth”)) OR (((((((Obstetric Labor, Premature[MeSH Terms]) OR Labor, Premature Obstetric[MeSH Terms]) OR Premature Labor[MeSH Terms]) OR Preterm Labor[MeSH Terms]) OR Labor, Preterm[MeSH Terms]) OR Labor, Premature[MeSH Terms]) OR Premature Obstetric Labor[MeSH Terms])) OR ((((((((Premature Birth[MeSH Terms]) OR birth, premature[MeSH Terms]) OR Births, Premature[MeSH Terms]) OR Premature Births[MeSH Terms]) OR Preterm Birth[MeSH Terms]) OR Birth, Preterm[MeSH Terms]) OR Births, Preterm[MeSH Terms]) OR Preterm Births[MeSH Terms]))) AND ((((((((((((“cervical length/transvaginal sonography”) OR “cervical length”) OR “short cervix”) OR “short cervix length”) OR “cervical shortening”) OR “ripening cervix”) OR “cervix insufficiency”)) OR ((((Cervical Ripening[MeSH Terms]) OR Cervical Ripenings[MeSH Terms]) OR Ripening, Cervical[MeSH Terms]) OR Ripenings, Cervical[MeSH Terms])) OR (((((((((Uterine Cervical Incompetence[MeSH Terms]) OR Cervical Incompetence, Uterine[MeSH Terms]) OR Incompetence, Uterine Cervical[MeSH Terms]) OR Cervix Incompetence[MeSH Terms]) OR Incompetence, Cervix[MeSH Terms]) OR Incompetent Cervix[MeSH Terms]) OR Cervices, Incompetent[MeSH Terms]) OR Cervix, Incompetent[MeSH Terms]) OR Incompetent Cervices[MeSH Terms])) OR (((((Cervix Uteri[MeSH Terms]) OR Cervixes[MeSH Terms]) OR Uterine Cervix[MeSH Terms]) OR Cervix, Uterine[MeSH Terms]) OR Cervix[MeSH Terms])) OR ((Cervical Length Measurement[MeSH Terms]) OR Cervical Length Measurements[MeSH Terms])).

EMBASE:

(‘cervical length measurement’/syn OR ‘short cervix’/syn OR ‘uterine cervix incompetence’/syn OR ‘uterine cervix ripening’/syn) AND (‘prematurity’/syn OR ‘premature labor’/syn) AND (‘prediction’/syn OR ‘screening’/syn OR ‘echography’/syn OR ‘transvaginal echography’/syn OR ‘second trimester pregnancy’/syn OR ‘second trimester pregnancy’/syn)
