# Supplementary material for: Cervical length varies considering different populations and gestational outcomes: Results from a systematic review and meta-analysis
Source: PLoS One. 2021 Feb 16;16(2):e0245746. doi: 10.1371/journal.pone.0245746 (PMC7886126; doi:10.1371/journal.pone.0245746)

**S3 Appendix – Women classification in high or low-risk for preterm birth**

All included articles collected information concerning risk of prematurity, however the classification in high or low-risk was not homogeneous along the included articles. High-risk could vary from minor Müllerian malformations and cervical excisional procedures, which may have no impact in cervical length, to a broad obstetrical history of preterm birth, ranging from extremely premature deliveries under 28 gestational weeks to as near term as 36 weeks. Our option was to maintain the original author´s description and define only two groups: one named as low-risk, which we believe to be more homogeneous, and a high-risk one, including a wide scale of history of prematurity risk as pointed above.

Perhaps all articles collected information concerning preterm birth risk, only 12 articles provided data on cervical measurement according to risk, totalizing 21,288 low-risk and 1,099 high-risk women. Although mean lengths of 33.94 (CI 95% 25.99-41.88) mm for high risk women *versus* 37.19 (CI 95% 33.81-40.56) mm for low risk seem clinically different, the confidence intervals values are large and overlap each other, prohibiting any inference regarding prematurity risk on cervical length measurement. Since the meta-analysis included less than 7% of all women (22,387 out of 322,656), this association still demands further studies. The follow figure shows forest plots of cervical length measurements of low and high risk women for preterm birth.


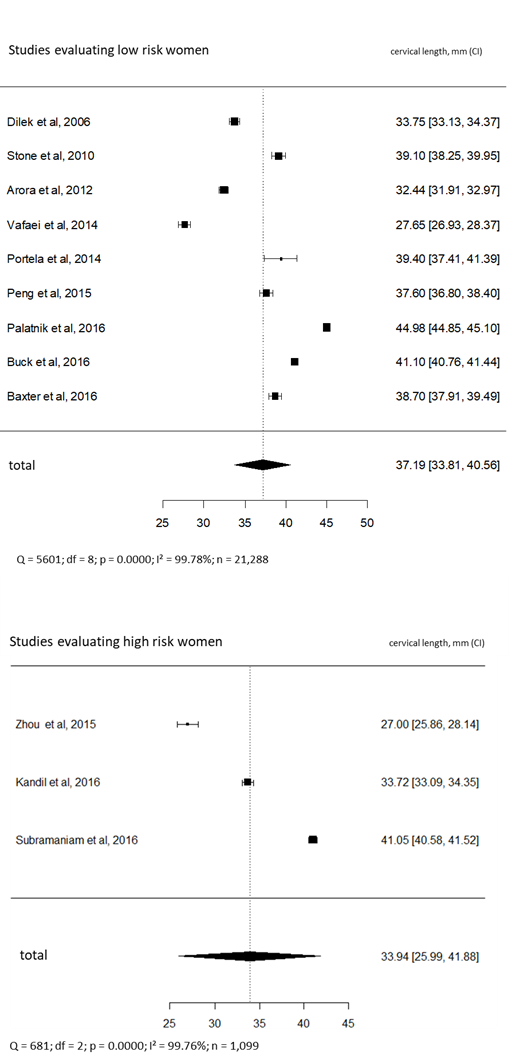

Supplement: S3 Appendix — (DOCX) [file pone.0245746.s003.docx]
