# Supplementary material for: Cervical length varies considering different populations and gestational outcomes: Results from a systematic review and meta-analysis
Source: PLoS One. 2021 Feb 16;16(2):e0245746. doi: 10.1371/journal.pone.0245746 (PMC7886126; doi:10.1371/journal.pone.0245746)
Supplement: S5 Appendix — (DOCX) [file pone.0245746.s005.docx]

**S5 Appendix - Risk of bias in included articles**

| article | *1- Clear question?* | *2- Well-defined and specified population?* | *3- Participation rate min 50%?* | *4- Same population and period?* | *5- Power description, or variance and effect estimates provided?* | *6- Exposure verified before outcome?* | *7- Timeframe sufficient exposure and outcome o?* | *8- Categories of exposure, or exposure measured as continuous variable?* | *9- Exposure measures clearly defined, valid, reliable, and consistent?* | *10* *exposure(s) assessed more than once?* | *11- Outcome measures clearly defined, valid, reliable, and consistent?* | *12- Outcome assessors blinded?* | *13* *follow-up after baseline 20% or less?* | *14- Potential confounding variables measured?* | *Total* |
| --- | --- | --- | --- | --- | --- | --- | --- | --- | --- | --- | --- | --- | --- | --- | --- |
| Iams et al, 1996**(12)** | Y | Y | Y | Y | Y | Y | Y | 2 | Y | Y | Y | Y | Y | N | 13 |
| Taipale & Hiilesmaa, 1998**(20)** | N | Y | Y | Y | N | Y | Y | 2 | Y | N | N | N | Y | Y | 9 |
| Hibbard et al, 2000**(93)** | Y | Y | Y | Y | N | Y | Y | 2 | Y | N | Y | N | Y | Y | 12 |
| Owen et al, 2001**(18)** | Y | Y | Y | Y | N | Y | Y | cont | Y | Y | Y | Y | Y | N | 11 |
| Iams et al, 2001**(94)** | Y | Y | Y | Y | N | Y | Y | cat | Y | N | Y | N | Y | N | 9 |
| To et al, 2001**(95)** | Y | Y | Y | Y | N | Y | Y | 2 | Y | Y | Y | N | Y | Y | 12 |
| Gramellini et al, 2002**(96)** | Y | Y | Y | Y | N | Y | Y | cont | Y | N | Y | N | Y | N | 9 |
| Fukami et al, 2003**(97)** | Y | Y | Y | Y | N | Y | Y | cont | Y | N | Y | N | Y | N | 9 |
| Carvalho et al, 2003**(98)** | Y | Y | Y | Y | N | Y | Y | cont | Y | Y | Y | N | Y | Y | 11 |
| Palma-Dias et al, 2004**(99)** | Y | Y | Y | Y | N | Y | Y | 2 | Y | N | Y | N | Y | Y | 11 |
| To et al, 2004**(100)** | Y | Y | N | Y | Y | Y | Y | cat | Y | N | Y | N | Y | N | 9 |
| Pires et al, 2004**(101)** | Y | Y | Y | Y | N | Y | Y | cat | Y | N | N | N | Y | N | 8 |
| Erasmus et al, 2005**(102)** | Y | Y | Y | Y | N | Y | Y | 2 | Y | Y | Y | N | Y | N | 11 |
| Leung et al, 2005**(103)** | Y | Y | Y | Y | Y | Y | Y | 2 | Y | N | Y | N | Y | Y | 11 |
| Durnwald et al, 2005**(104)** | Y | Y | Y | Y | N | Y | Y | cat | Y | Y | N | N | Y | Y | 10 |
| de Carvalho et al, 2005**(105)** | Y | Y | Y | Y | N | Y | Y | 2 | Y | N | Y | N | Y | Y | 11 |
| Matijevic et al, 2006**(106)** | Y | Y | Y | Y | Y | Y | Y | 2 | Y | N | Y | Y | Y | N | 12 |
| Dilek et al, 2006**(107)** | Y | Y | Y | Y | Y | Y | Y | cont | Y | N | Y | N | Y | N | 10 |
| To et al, 2006**(108)** | Y | Y | Y | Y | Y | Y | Y | 2 | Y | N | Y | N | Y | N | 11 |
| Brandão et al, 2006**(109)** | N | Y | Y | Y | N | Y | Y | cont | Y | N | N | N | Y | N | 7 |
| Ozdemir et al, 2007**(110)** | Y | Y | Y | Y | N | Y | Y | cont | Y | Y | Y | N | Y | Y | 11 |
| Salomon et al, 2009**(111)** | Y | Y | Y | Y | Y | Y | Y | 2 | Y | Y | Y | Y | Y | N | 13 |
| Stone et al, 2010**(112)** | Y | Y | Y | Y | N | Y | Y | 2 | Y | N | N | N | Y | N | 9 |
| Owen et al, 2010**(113)** | Y | Y | Y | Y | N | Y | Y | 2 | Y | Y | Y | N | Y | Y | 12 |
| Donders et al, 2010**(114)** | Y | Y | Y | Y | N | Y | Y | cont | Y | Y | Y | N | N | N | 9 |
| Silva et al, 2010**(115)** | Y | Y | Y | Y | N | Y | Y | 2 | Y | Y | Y | N | Y | N | 11 |
| Barber et al, 2010**(116)** | Y | Y | Y | Y | N | Y | Y | cont | Y | Y | Y | N | Y | N | 10 |
| Stǎnescu et al, 2010 | Y | Y | Y | Y | N | Y | Y | cont | Y | N | Y | N | Y | N | 9 |
| Park et al, 2011**(118)** | N | Y | Y | Y | N | Y | Y | 2 | Y | N | N | N | Y | Y | 9 |
| Souka et al, 2011**(119)** | Y | Y | Y | Y | Y | Y | Y | cont | Y | Y | Y | N | Y | Y | 12 |
| Hassan et al, 2011**(16)** | Y | Y | Y | Y | Y | Y | Y | cat | Y | N | Y | N | Y | Y | 11 |
| Qu et al, 2011 **(120)** | Y | Y | Y | Y | N | Y | Y | cont | Y | Y | Y | N | Y | N | 10 |
| Arora et al, 2012**(121)** | Y | Y | Y | Y | N | Y | Y | 2 | Y | N | N | N | Y | N | 9 |
| Barber et al, 2012**(122)** | N | Y | Y | Y | N | Y | Y | cont | Y | N | N | N | Y | N | 7 |
| Goya et al, 2012**(123)** | Y | Y | Y | Y | Y | Y | Y | 2 | Y | N | N | N | Y | Y | 11 |
| Dalili et al, 2013**(124)** | Y | Y | Y | Y | Y | Y | Y | 2 | Y | N | N | N | Y | N | 10 |
| Facco & Simhan, 2013**(125)** | Y | Y | Y | Y | N | Y | Y | cont | Y | N | Y | N | Y | N | 9 |
| Friedman et al, 2013**(126)** | Y | Y | Y | Y | N | Y | Y | 2 | Y | N | Y | N | Y | N | 10 |
| D´Agostini et al, 2013**(127)** | Y | Y | Y | Y | Y | Y | Y | 2 | Y | N | Y | N | Y | N | 11 |
| Borna et al, 2013**(128)** | Y | Y | Y | Y | N | Y | Y | cont | Y | N | N | N | Y | N | 8 |
| Mella et al, 2013**(129)** | Y | Y | Y | Y | N | Y | Y | 2 | Y | N | N | N | Y | Y | 10 |
| Hui et al, 2013**(130)** | Y | Y | Y | Y | Y | Y | Y | cat | Y | N | Y | Y | N | N | 9 |
| Orzechowski et al, 2014**(131)** | N | Y | Y | Y | Y | Y | Y | cat | Y | N | Y | N | N | N | 8 |
| Vafaei et al, 2014**(132)** | N | Y | N | Y | N | Y | Y | cont | Y | N | Y | N | Y | N | 7 |
| Portela et al, 2014**(133)** | Y | Y | Y | Y | Y | Y | Y | cont | Y | N | Y | N | Y | Y | 11 |
| Miller & Grobman, 2014**(134)** | Y | Y | Y | Y | Y | Y | Y | cont | Y | N | Y | N | Y | Y | 11 |
| Miller et al, 2015**(135)** | Y | Y | Y | Y | N | Y | Y | cat | Y | N | Y | N | Y | N | 9 |
| van der Ven et al, 2015**(136)** | Y | Y | Y | Y | N | Y | Y | 2 | Y | N | Y | N | N | Y | 10 |
| Palatnik & Grobman, 2015**(137)** | Y | Y | Y | Y | N | Y | Y | 2 | Y | N | Y | N | Y | Y | 11 |
| Peng et al, 2015**(138)** | Y | Y | Y | Y | N | Y | Y | 2 | Y | N | Y | N | Y | N | 10 |
| van der Ven et al, 2015**(139)** | Y | Y | Y | Y | N | Y | Y | 2 | Y | N | Y | N | Y | Y | 11 |
| Kuusela et al, 2015**(140)** | Y | Y | Y | Y | Y | Y | Y | 2 | Y | N | Y | N | Y | Y | 12 |
| Zhou et al, 2015**(141)** | Y | Y | Y | Y | Y | Y | Y | 2 | Y | N | Y | N | Y | N | 11 |
| Kandil et al, 2016**(142)** | Y | Y | Y | Y | N | Y | Y | 2 | Y | N | Y | N | N | Y | 10 |
| Puttanavijarn & Phupong, 2016**(143)** | Y | Y | Y | Y | N | Y | Y | cont | Y | N | Y | N | Y | N | 9 |
| Palatnik et al, 2016**(144)** | Y | Y | Y | Y | N | Y | Y | 2 | Y | N | Y | N | Y | Y | 11 |
| Buck et al, 2016**(145)** | Y | Y | Y | Y | N | Y | Y | 2 | Y | N | Y | N | Y | Y | 11 |
| Kongwattanakul et al, 2016**(146)** | N | Y | Y | Y | Y | Y | Y | 2 | Y | N | N | N | Y | Y | 10 |
| Jwala et al, 2016**(147)** | Y | Y | Y | Y | Y | Y | Y | 2 | Y | N | Y | Y | Y | Y | 13 |
| Son et al, 2016**(148)** | Y | Y | Y | Y | Y | Y | Y | cat | Y | N | Y | N | Y | Y | 11 |
| Baxter et al, 2016**(149)** | Y | Y | Y | Y | Y | Y | Y | 2 | Y | Y | Y | Y | N | N | 12 |
| Temming et al, 2016**(150)** | Y | Y | Y | Y | N | Y | Y | cat | Y | N | Y | N | N | Y | 9 |
| Subramaniam et al, 2016**(151)** | Y | Y | Y | Y | Y | Y | Y | 2 | Y | Y | Y | N | N | N | 11 |
| Baños et al, 2017**(152)** | Y | Y | Y | Y | Y | Y | Y | 2 | Y | N | Y | Y | Y | N | 12 |
| Esplin et al, 2017**(153)** | Y | Y | Y | Y | Y | Y | Y | cat | Y | Y | Y | Y | Y | Y | 13 |
| van Os et al, 2017**(154)** | Y | Y | Y | Y | Y | Y | Y | 2 | Y | N | Y | N | Y | Y | 12 |
| Buck et al, 2017**(61)** | Y | Y | Y | Y | N | Y | Y | 2 | Y | N | Y | N | Y | Y | 11 |
| Kazemier et al, 2017**(155)** | Y | Y | Y | N | N | Y | Y | 2 | Y | N | Y | N | Y | Y | 10 |
| Harville et al, 2017**(156)** | Y | Y | Y | Y | N | Y | Y | cat | Y | Y | Y | Y | Y | Y | 12 |
| Hermans et al, 2017(12358) | Y | Y | Y | Y | N | Y | Y | 2 | Y | Y | Y | N | Y | Y | 12 |
| Liff I et al, 2020(179) | Y | Y | Y | Y | Y | Y | Y | cat | Y | Y | Y | N | Y | Y | 13 |
| Wongkanha et al, 2020(256) | Y | Y | Y | Y | N | Y | Y | cat | Y | N | Y | Y | Y | Y | 12 |
| Mishra et al, 2018(147) | Y | Y | Y | Y | Y | Y | Y | cat | Y | N | Y | N | Y | N | 11 |
| Maerdan et al, 2017(25328) | Y | Y | Y | Y | N | Y | Y | cat | Y | N | Y | N | Y | N | 10 |
| Marinelli et al, 2020(248) | Y | Y | Y | Y | N | Y | Y | cat | Y | N | Y | N | Y | Y | 11 |
| Peixoto et al, 2017(751) | Y | Y | Y | Y | N | Y | Y | cat | Y | N | Y | N | Y | N | 10 |
| Farràs et al, 2020(1413) | Y | Y | Y | Y | Y | Y | Y | cat | Y | Y | Y | N | Y | Y | 13 |

cont = continuous variable; cat = categorical variable 2 = continuous + categorical variable
