# Supplementary material for: Cervical length varies considering different populations and gestational outcomes: Results from a systematic review and meta-analysis
Source: PLoS One. 2021 Feb 16;16(2):e0245746. doi: 10.1371/journal.pone.0245746 (PMC7886126; doi:10.1371/journal.pone.0245746)
Supplement: S3 Fig — Comparison of cervical length measurements in adolescents and adults. (DOCX) [file pone.0245746.s008.docx]

**S3 Fig - Adolescents versus adults**


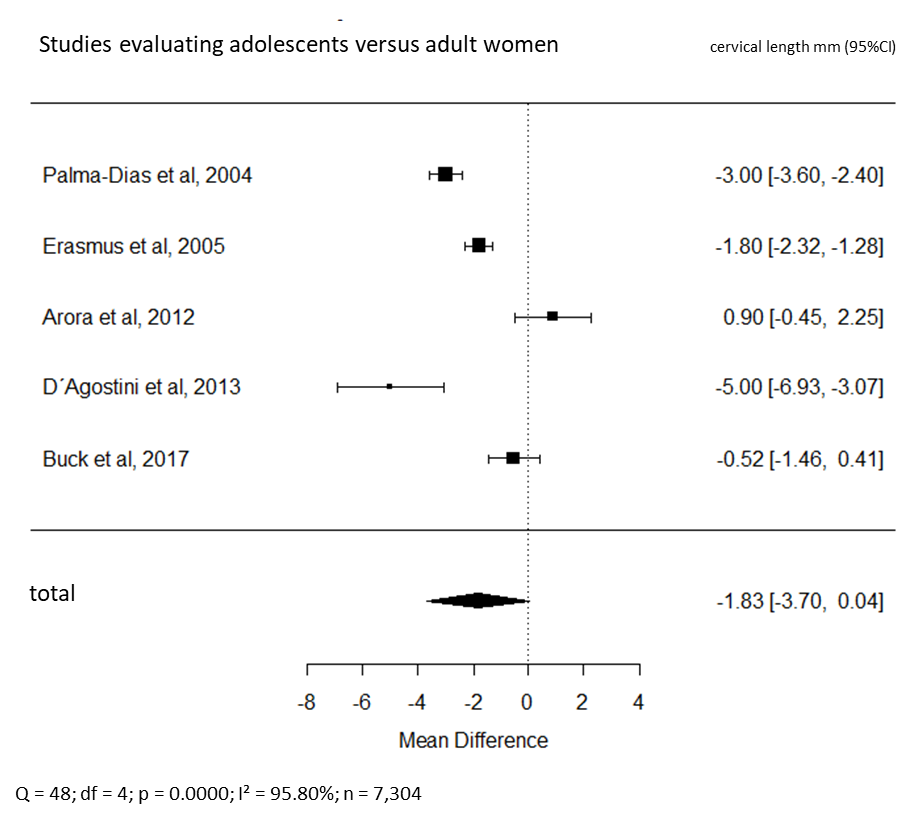


Comparison of cervical length measurements in adolescents and adults.
