# Supplementary material for: Cervical length varies considering different populations and gestational outcomes: Results from a systematic review and meta-analysis
Source: PLoS One. 2021 Feb 16;16(2):e0245746. doi: 10.1371/journal.pone.0245746 (PMC7886126; doi:10.1371/journal.pone.0245746)
Supplement: S1 Table — (DOCX) [file pone.0245746.s009.docx]

**S1 Table – Excluded articles classified according to exclusion reasons**

| **Publication** | **Risk** | **Gestational age (weeks + days)** | **n** | **Reason for exclusion** |
| --- | --- | --- | --- | --- |
| **Zorzoli et al, 1994 (Italy)** (30) | general | 12-31 | 154 | Gestational age surpassing review´s scope |
| **Carr et al, 2000 (USA)** (31) | high | 14-40 | 84 |  |
| **Cook et al, 2000 (Australia)** (32) | high | 9-29 | 81 |  |
| **Stevens-Simon et al, 2000 (USA)** (33) | general | 18-29+6 | 46 |  |
| **Macdonald et al, 2001 (UK)** (34) | high | 12-41 | 106 |  |
| **Guzman et al, 2001 (USA) (19)** | high | 15-24 | 469 |  |
| **Berghella et al, 2004 (USA)** (35) | high | 14-23 | 319 |  |
| **Cheng , 2006 (China)** (36) | general | 8-42 | 61 |  |
| **Aboulghar et al, 2009 (Egypt)** (37) | low | NR* | 153 |  |
| **Marsosi et al, 2010 (Iran)** (38) | high | 14-28 | 200 |  |
| **Şendaǧ et al, 2010 (Turkey)** (39) | general | 20-26 | 207 |  |
| **Souka et al, 2011 (Greece)** (40) | general | 11-24 | 978 |  |
| **Hernandez-Andrade et al, 2012 (USA)** (41) | general | 6+2-39 | 220 |  |
| **Moroz et al, 2012 (USA)** (42) | general | 21-28 | 2,695 |  |
| **Chaudhury et al, 2013 (India)** (43) | low | 18-26 | 127 |  |
| **Roh et al, 2013 (South Korea)** (44) | general | 20-29 | 307 |  |
| **Hatanaka et al, 2014 (Brazil)** (45) | high | 16 a 26 | 159 |  |
| **Çakıroğlu et al, 2015 (Turkey)** (46) | general | 11-24 | 235 |  |
| **Cho et al, 2015 (South Korea)** (47) | general | 20-29 | 771 |  |
| **Findley et al, 2015 (USA)** (48) | general | 15 a 26 | 1,074 |  |
| **Papastefanou et al, 2016 (Greece)** (49) | general | 11-40 | 4,397 |  |
| **Hughes et al, 2017 (Australia)** (50) | high | 14-26 | 756 |  |
| **Price et al, 2019 (Zambia)** | general | 16-28 | 1171 |  |
| **Cobaleda et al, 2019 (Spain)** | General | 18-33+6 | 57 |  |
| **Maia et al, 2019 (Brazil)** | high | 25-34+6 | 95 |  |
| **Thain et al, 2020 (Singapore)** | General | <14 | 926 |  |
| **Andrade et al, 2017 (Brazil)** | general | <16 | 38 |  |
| **Tsakiridis et al, 2019 (Greece)** | general | 31-34 | 240 |  |
| **Heath et al, 1998 (UK)** (51) | general | 22-24 | 2,702 | Cohort already included |
| **Heath et al, 1998 (UK)**(52) | general | 22-24 | 2,702 |  |
| **Heath et al, 2000 (UK)**(53) | general | 22-24 | 24 |  |
| **Mercer et al, 2000 (USA)**(54) | general | 23-24 | 2,929 |  |
| **Owen et al, 2004 (USA)**(55) | high | 16-23+6 | 183 |  |
| **Yost et al, 2004 (USA)**(56) | high | 16-23+6 | 181 |  |
| **Berghella et al, 2007 (USA)(11)** | high | 16-23+6 | 183 |  |
| **Dilek et al, 2007 (Turkey)**(57) | general | 16-24 | 257 |  |
| **Owen et al, 2009 (USA)**(58) | high | 16-21 | 1,014 |  |
| **Szychowski et al, 2009 (USA)**(59) | high | 16-22+6 | 1,014 |  |
| **Orzechowski et al, 2015 (USA)**(60) | general | 18-23+6 | 1,551 |  |
| **Van Os et al, 2015 (Netherlands)**(61) | low | 18-22 | 20,234 |  |
| **Buck et al, 2017 (USA)**(62) | low | 18-23+6 | 1,751 |  |
| **Baños et al, 2017 (Spain)**(63) | general | 19-24 | 310 | Nested case-control |
| **Orion et al, 2001 (USA)**(64) | general | 16-24 | NR* | Does not describe total of screened women |
| **Althuisius et al, 2003 (Netherlands)**(65) | high | < 27 |  |  |
| **Arabin et al, 2003 (Germany and Netherlands)**(66) | high | < 24 |  |  |
| **Hassan et al, 2007 (USA)**(67) | high | 14-24 |  |  |
| **O’Brien et al, 2007 (multicentric)**(68) | general | 16-22 |  |  |
| **Keeler et al, 2009 (USA)** (69) | high | 16-24 |  |  |
| **Barinov et al, 2016 (Russia)**(70) | high | < 24 |  |  |
| **Nicolaides et al, 2016 (UK, Slovenia, Portugal, Chile, Australia, Italy, Albania, GermanyandBelgium)**(16) | general | 20-24 |  |  |
| **Norman et al, 2016 (UK and Sweden)**(71) | high | 18-24 |  |  |
| **Saccone et al, 2017 (Italy)**(72) | high | 18-23+6 |  |  |
| **Guzman et al, 1998 (USA)**(73) | general | 15-24 | 89 | different ultrasound technique |
| **Guzman et al, 1998 (USA)** (74) | general | 15-24 | 155 |  |
| **Bloechle et al, 1994(Germany)**(75) | general | 20-23 | 115 |  |
| **To et al, 2000 (UK)**(76) | general | 23 | 149 |  |
| **Iams et al, 2011 (USA)**(77) | general | 22-24+6 | 2,521 |  |
| **Tanvir et al, 2014 (India)**(78) | general | 22-24 | 130 |  |
| **Foroozanfard et al, 2015 (Iran)**(79) | general | 18-24 | 438 |  |
| **Pandipati et al, 2015 (Australia)**(80) | general | 18-23+6 | 1,580 |  |
| **Yilmaz Dogru et al, 2016 (Turkey)**(81) | general | 18-22 | 148 |  |
| **Garthey et al, 2018 (USA)** | general | 18-23+6 | 1348 |  |
| **Venkatesh et al, 2018 (USA)** | high | 14-22+6 | 356 |  |
| **Yan et al, 2019 (China)** | general | 12-20 | 3688 |  |
| **Park et al, 2020 (Korea)** | general | 18-22 | 537 |  |
| **Thangaraj et al, 2018 (India)** | general | 20-24 | 173 |  |
| **Puttanavijarn et al, 2016 (Thailand)** | general | 16-23+6 | 160 |  |
| **Dowd et al, 2001 (Australia)**(82) | general | 15-24 | 302 | Exclusion criteria: symptoms, only preterm, only altered cervices, twins |
| **Hoesli et al, 2003 (Switzerland)** (83) | general | 20-33+6 | 669 |  |
| **Hebbar & Samjhana, 2006 (Malaysia)**(84) | low | 20-24 | 168 |  |
| **Fonseca et al, 2007 (UK, Chile, Brazil and Greece) (5)** | general | 20-25 | 23,795 |  |
| **Itaborahy et al, 2010 (Brazil)**(85) | general | 20-34 | 145 |  |
| **Broumand et al, 2011 (Iran)**(86) | high | 13-18 | 56 |  |
| **Pessel et al, 2013 (USA)**(87) | high | 16-32 | 237 |  |
| **Banicevic et al, 2014 (Bosnia and Herzegovina)**(88) | general | 16-37 | 102 |  |
| **Wozniak et al, 2014 (Poland)**(89) | general | 18-22 | 333 |  |
| **Palatnik et al, 2015 (USA)**(90) | high | 18-22 | 1,536 |  |
| **Suhag et al, 2015 (USA)**(91) | high | < 24 | 102 |  |
| **Gimovsky et al, 2016 (USA)**(92) | high | 15-24 | 112 |  |
| **Weichert et al, 2016 (Germany)**(93) | general | 22-33+6 | 36 |  |
| **Dugoff et al, 2017 (USA)** | high | 18-23+6 | 242 |  |
| **Manzour et al, 2019 (Spain)** | high | 19-22 | 131 |  |
| **Poojari et al, 2019 (India)** | general | NR | 228 |  |
| Berghella et al, 2017 (USA) | high | 18-27+6 | 46 |  |
| Price et al, 2020 (Zambia | general | < 24 | 140 |  |

*NR – not reported
