# Supplementary material for: Cervical length varies considering different populations and gestational outcomes: Results from a systematic review and meta-analysis
Source: PLoS One. 2021 Feb 16;16(2):e0245746. doi: 10.1371/journal.pone.0245746 (PMC7886126; doi:10.1371/journal.pone.0245746)
Supplement: S2 Table — (DOCX) [file pone.0245746.s010.docx]

**S2 Table - Main characteristics of included articles**

| **article** | **Country** | **Method** | **Population** | **Gestational age (weeks + days)** | **n** | **Results reported** |
| --- | --- | --- | --- | --- | --- | --- |
| **Iams et al, 1996**(12) | USA | cohort | general risk | 22-24 | 2,915 | mean,  percentile |
| **Taipale & Hiilesmaa, 1998**(20) | Finland | cohort | general risk | 18-22+6 | 3,694 | mean,  n in stratum |
| **Hibbard et al, 2000**(94) | USA | cohort | general risk | 16-22 | 760 | mean  n in stratum |
| **Owen et al, 2001**(18) | USA | cohort | with previous preterm | 16-23+6 | 183 | percentile,  n in stratum |
| **Iams et al, 2001**(95) | USA | cohort | general risk | 22-24 | 2,197 | n in stratum |
| **To et al, 2001**(96) | UK | clinical trial | general risk | 22-24 | 6,819 | mean, percentile,  n in stratum |
| **Gramellini et al, 2002**(97) | Italy | cross sectional | general risk | 20-25 | 321 | mean,  by GA* |
| **Fukami et al, 2003**(98) | Japan | cohort | general risk | 16-19 | 3,030 | mean |
| **Carvalho et al, 2003**(99) | Brazil | cohort | general risk | 22-24 | 529 | mean |
| **Palma-Dias et al, 2004**(100) | Brazil | cohort | general risk | 22-24 | 1,131 | mean, percentile,  n in stratum |
| **To et al, 2004**(101) | UK, Brazil, Slovenia, South Africa, Greece and Chile | clinical trial | general risk | 22-24 | 47,123 | n in stratum |
| **Pires et al, 2004**(102) | Brazil | cohort | general risk | 21-24 | 361 | n in stratum |
| **Erasmus et al, 2005**(103) | South Africa | cohort | general risk | 23-23+6 | 1,603 | mean,  n in stratum |
| **Leung et al, 2005**(104) | Hong Kong | cohort | general risk | 18-22 | 2,880 | mean,  percentile |
| **Durnwald et al, 2005**(105) | USA | cohort | with previous preterm | 22-24+6 | 188 | n in stratum |
| **de Carvalho et al, 2005**(106) | Brazil | cohort | general risk | 21-24 | 1,958 | mean,  n in stratum |
| **Matijevic et al, 2006**(107) | Croácia | clinical trial | nulliparous, general risk | 16-23 | 138 | mean,  percentile |
| **Dilek et al, 2006**(108) | Turquia | cohort | with previous preterm | 22-22+6 | 250 | mean |
| **To et al, 2006**(109) | UK | clinical trial | general risk | 22-24+6 | 39,284 | mean,  n in stratum |
| **Brandão et al, 2006**(110) | Brazil | cohort | general risk | 19-24 | 74 | mean |
| **Ozdemir et al, 2007**(111) | Turquia | cohort | general risk | 20-24 | 152 | mean |
| **Salomon et al, 2009**(112) | France, Spain | cohort | general risk | 16-36 | 6,614 | mean,  by GA*,  percentile,  n in stratum |
| **Stone et al, 2010**(113) | Australia, New Zealand | cohort | nulliparous, general risk | 18-20 | 203 | mean,  percentile |
| **Owen et al, 2010**(114) | USA | clinical trial | with previous preterm | 16-21+6 | 1,014 | mean,  n in stratum |
| **Donders et al, 2010**(115) | Belgium | cohort | general risk | 20-24 | 983 | mean |
| **Silva et al, 2010**(116) | Brazil | cohort | general risk | 20-24 | 743 | by GA*,  percentile |
| **Barber et al, 2010**(117) | Spain | cohort | general risk | 18-22 | 2,351 | mean |
| **Stǎnescu et al, 2010** (118) | Romenia | cohort | low risk | 18-20 | 471 | mean |
| **Park et al, 2011**(119) | South Korea | cohort | general risk | 20-24 | 374 | mean,  percentile |
| **Souka et al, 2011**(120) | Greece | cohort | general risk | 16-24 | 800 | mean |
| **Hassan et al, 2011**(15) | USA | clinical trial | general risk | 19-23+6 | 32,091 | n in stratum |
| **Qu et al, 2011** (121) | China | cohort | general risk | 22-24 | 5,277 | mean |
| **Arora et al, 2012**(122) | India | cohort | low risk | 20-24 | 200 | mean,  n in stratum |
| **Barber et al, 2012**(123) | Spain | cohort | low risk | 20-22 | 306 | mean |
| **Goya et al, 2012**(124) | Spain | clinical trial | general risk | 18-22 | 11,294 | mean,  n in stratum |
| **Dalili et al, 2013**(125) | Iran | cohort | general risk | 21-24 | 450 | mean,  n in stratum |
| **Facco & Simhan, 2013**(126) | USA | cohort | general risk | 22-24 | 3,056 | n in stratum |
| **Friedman et al, 2013**(127) | USA | cohort | high risk | 18-23+6 | 703 | mean,  n in stratum |
| **D´Agostini et al, 2013**(128) | Brazil | cross sectional | nulliparous, general risk | 21-24 | 80 | mean,  n in stratum |
| **Borna et al, 2013**(129) | Iran | cohort | general risk | 15-23 | 86 | mean |
| **Mella et al, 2013**(130) | USA | cohort | general risk | 18-23+6 | 639 | mean,  n in stratum |
| **Hui et al, 2013**(131) | China | clinical trial | low risk | 20-24 | 4,438 | n in stratum |
| **Orzechowski et al, 2014**(132) | USA | cohort | no previous preterm, general risk | 18-23+6 | 1,569 | n in stratum |
| **Vafaei et al, 2014**(133) | Iran | cohort | low risk | 18-24 | 195 | mean |
| **Portela et al, 2014**(134) | Brazil | cohort | low risk | 22-24 | 56 | mean |
| **Miller & Grobman, 2014**(135) | USA | cohort | general risk | 18-24 | 6,209 | mean |
| **Miller et al, 2015**(136) | USA | cohort | general risk | 18-23+6 | 18,250 | n in stratum |
| **van der Ven et al, 2015**(137) | Netherlands | cohort | general risk | 16-21+6 | 11,943 | mean,  percentile,  n in stratum |
| **Palatnik & Grobman, 2015**(138) | USA | cohort | general risk | 18-22+6 | 1,024 | mean,  n in stratum |
| **Peng et al, 2015**(139) | Taiwan | cohort | no previous preterm, general risk | 20-24 | 174 | mean,  percentile,  n in stratum |
| **van der Ven et al, 2015**(140) | Netherlands and Australia | cohort | general risk | 18-22+6 | 5,092 | percentile,  n in stratum |
| **Kuusela et al, 2015**(141) | Sweden | cohort | general risk | 16-23+1 | 2,122 | mean,  by GA*,  percentile,  n in stratum |
| **Zhou et al, 2015**(142) | China | cohort | high risk | 14-22 | 213 | n in stratum  mean |
| **Kandil et al, 2016**(143) | Egypt | cohort | with previous preterm | 20-22+6 | 100 | mean,  n in stratum |
| **Puttanavijarn & Phupong, 2016**(144) | Thailand | cohort | general risk | 16-23+6 | 160 | mean |
| **Palatnik et al, 2016**(145) | USA | cohort | low risk | 18-24 | 18,100 | mean,  n in stratum |
| **Buck et al, 2016**(146) | USA | cohort | low risk | 18-23+6 | 1,751 | mean,  n in stratum |
| **Kongwattanakul et al, 2016**(147) | Thailand | cohort | general risk | 18-23 | 307 | mean,  n in stratum |
| **Jwala et al, 2016**(148) | USA | cohort | general risk | 18-23+6 | 528 | mean,  n in stratum |
| **Son et al, 2016**(149) | USA | cohort | general risk | 18-24 | 17,590 | n in stratum |
| **Baxter et al, 2016**(150) | USA | clinical trial | low risk | 17-23 | 359 | mean,  n in stratum |
| **Temming et al, 2016**(151) | USA | cohort | general risk | 17-23+6 | 10,871 | n in stratum |
| **Subramaniam et al, 2016**(152) | USA |  | with previous preterm | 16-22+6 | 786 | mean,  n in stratum |
| **Baños et al, 2017**(153) | Spain | cohort | no previous preterm, low risk | 19-24 | 532 | mean,  by GA*,  n in stratum |
| **Esplin et al, 2017**(154) | USA | cohort | nulliparous, general risk | 16-22+6 | 9,469 | n in stratum |
| **van Os et al, 2017**(155) | Netherlands | cohort | general risk | 18-22 | 20,234 | mean,  n in stratum |
| **Buck et al, 2017**(62) | USA | cohort | low risk | 18-23+6 | 341 | mean,  n in stratum |
| **Kazemier et al, 2017**(156) | USA and Netherlands | cohort | nulliparous, low risk | 16-21 | 6,743 | mean,  percentile,  n in stratum |
| **Harville et al, 2017**(157) | USA | cohort | with previous preterm | 16-24 | 175 | n in stratum |
| **Hermans et al, 2017(12358)** | Netherlands | cohort | no previous preterm | 16-22 | 12358 | median , n in stratum |
| **Liff I et al, 2020(179)** | Botswana | cohort | general risk | 12-24+6 | 179 | mean, median, n in stratum |
| **Wongkanha et al, 2020(256)** | Thailand | cross sectional | no with previous preterm | 16-24 | 256 | mean, n in stratum |
| **Mishra et al, 2018(147)** | India | cohort | no with previous preterm | 16-24 | 147 | mean |
| **Maerdan et al, 2017(25328)** | China | cohort | general risk | 20-24 | 25328 | median, n in stratum |
| **Marinelli et al, 2020(248)** | Brazil | cross sectional | general risk | 20-23+6 | 248 | n in stratum |
| **Peixoto et al, 2017(751)** | Brazil | cohort | general risk | 20-24+6 | 751 | mean |
| **Farràs et al, 2020(1413)** | Barcelona | cohort | general risk | 19-22+6 | 1413 | mean, median, n in stratum |

*GA = gestational age
