# Supplementary material for: Cervical length varies considering different populations and gestational outcomes: Results from a systematic review and meta-analysis
Source: PLoS One. 2021 Feb 16;16(2):e0245746. doi: 10.1371/journal.pone.0245746 (PMC7886126; doi:10.1371/journal.pone.0245746)
Supplement: Attachment — Submitted filename: Response to the reviewers_rev2.docx [file pone.0245746.s012.docx]

Dear Editor, Dr.Frank T. Spradley

We would like to thank you and the reviewer for the careful reading of our updated article. Below we discuss the reviewer comment.

Thank you for consider our study and we are available for more clarifications.

With kind regards,

Rodolfo Pacagnella

Review Comments to the Author and Answer

Reviewer #2: I am happy with the changes made by the authors. I wonder however, as the search was extended to July 2020 why the authors did not consider the study by Souka et al presenting the results of screening in a routine obstetric population (Souka AP, Papastefanou I, Pilalis A, Kassanos D, Papadopoulos G. Implementation of universal screening for preterm delivery by mid-trimester cervical-length measurement. Ultrasound Obstet Gynecol. 2019 Mar;53(3):396-401).

Thank you for the careful reading. We appreciate the highlight on this and possibility to discuss.

Souka et al. presented in 2019 a very interesting study regarding the implementation of transvaginal ultrasound as a screening test for preventing preterm birth, evolving a sample of 10,506 women. The study has very good quality and describes the cervix measurement technique adequately. In the results, this study describes the number of women considering different cutoff points for cervical length (≤10, ≤15 and ≤25mm) and two Gaussian curves to describe the distribution of cervical-length (CL) measurements in women who delivered spontaneously before 34 weeks and in those who delivered at term.

This study was retrieved by our search strategy, however, we did not consider the study into our metanalysis because the results presented by Souka et al. are not presented as mean and SD for the entire studied population. The authors describe cervical length distribution using Gaussian curve technique, which unfortunately makes it not possible to use the data necessary to be included in our meta-analysis. The authors only present mean cervical length by subgroups of cervical length (‘short’ and ‘long’) and gestational age at birth. Presented like this, the results could not be included in the metanalysis in which we used aggregated data. This study is a very important study and should be included in a further IPD metanalysis, however, we were unable to use the data into our analysis.
